# Interactions between long interpregnancy interval and advanced maternal age on neonatal outcomes

**DOI:** 10.1007/s12519-023-00728-4

**Published:** 2023-04-26

**Authors:** Yan Ma, Hua Fu, Yang li, Zheng-Rong Bao, Wen-Bin Dong, Xiao-Ping Lei

**Affiliations:** 1 Division of Neonatology, Department of Pediatrics, The Affiliated Hospital of Southwest Medical University, Xiaoping Lei, 8 Kangcheng Road, Luzhou, 646000 China; 2 Department of Perinatology, The Affiliated Hospital of Southwest Medical University, Luzhou, China; 3 Sichuan Clinical Research Center for Birth Defects, Luzhou, China; 4 School of Pediatrics, Southwest Medical University, Luzhou, China

**Keywords:** Interaction, Interpregnancy interval, Low birth weight, Maternal age, Preterm birth

## Abstract

**Background:**

After the implementation of the universal two-child policy in China, it was more frequent to have long interpregnancy intervals (IPIs) and advanced maternal age. However, the interactions between long IPIs and advanced maternal age on neonatal outcomes are unknown.

**Methods:**

The study subjects of this historical cohort study were multiparas with singleton live births between October 1st, 2015, and October 31st, 2020. IPI was defined as the interval between delivery and conception of the subsequent pregnancy. Logistic regression models were used to calculate adjusted odds ratios (aORs) and 95% confidence intervals (CIs) of the risks of preterm birth (PTB), low birth weight (LBW), small for gestation age, and 1-min Apgar score ≤ 7 in different IPI groups. Relative excess risk due to interaction (RERI) was used to evaluate the additive interaction between long IPIs and advanced maternal age.

**Results:**

Compared with the 24 ≤ IPI ≤ 59 months group, the long IPI group (IPI ≥ 60 months) was associated with a higher risk of PTB (aOR, 1.27; 95% CI: 1.07–1.50), LBW (aOR, 1.32; 95% CI 1.08–1.61), and one-minute Apgar score ≤ 7 (aOR, 1.46; 95% CI 1.07–1.98). Negative additive interactions (all RERIs < 0) existed between long IPIs and advanced maternal age for these neonatal outcomes. Meanwhile, IPI < 12 months was also associated with PTB (aOR, 1.51; 95% CI 1.13–2.01), LBW (aOR, 1.50; 95% CI 1.09–2.07), and 1-min Apgar score ≤ 7 (aOR, 1.93; 95% CI 1.23–3.04).

**Conclusions:**

Both short and long IPIs are associated with an increased risk of adverse neonatal outcomes. Appropriate IPI should be recommended to women planning to become pregnant again. In addition, better antenatal care might be taken to balance the inferiority of advanced maternal age and to improve neonatal outcomes.

**Supplementary Information:**

The online version contains supplementary material available at 10.1007/s12519-023-00728-4.

## Introduction

Birth outcomes are critical indicators for predicting infant health [1]. Adverse birth outcomes are related to health problems later in life and are important public health problems globally [2]. Interpregnancy intervals (IPIs) were identified as a key and potentially modifiable risk factor for adverse maternal and neonatal outcomes [3–6]. Previous studies revealed a J-shaped relationship between IPIs and adverse perinatal outcomes; that is, both short and long IPIs were associated with adverse perinatal outcomes [7–9]. Many previous studies [10–16] observed that a short IPI was a risk factor for adverse neonatal outcomes, including preterm birth (PTB), low birth weight (LBW), and small-for-gestational-age (SGA). Nevertheless, only a few studies have focused on the relationship between a long IPI and neonatal outcomes [10, 16].

Since the family planning policy was implemented in the 1980s in China, the one-child policy has been successively implemented in the past few decades. However, to maintain the growth rate of the population, the two-child policy was universally carried out in 2015, which allows all families to have a second child [17]. From the one-child policy to the universal two-child policy, many couples in China with only one child gave birth to their second child, which resulted in a significant increase in pregnant women with long IPIs. It provides a unique opportunity to study the association between long IPIs and neonatal outcomes during the period of family planning updates in China.

**Fig. 1:**
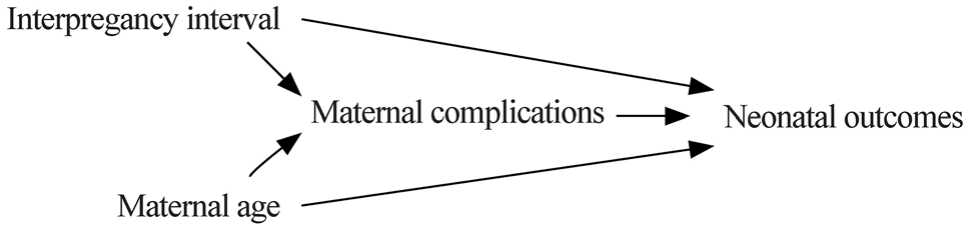
Directed acyclic graph (DAG)

Long IPIs are frequently combined with advanced maternal age (≥ 35 years), which is also associated with increased risks of adverse neonatal outcomes [18, 19]. Accompanied by the implementation of the universal two-child policy in 2015 in China, the rate of pregnant women with advanced maternal age increased significantly [20]. In general, advanced maternal age was also associated with a higher rate of maternal complications, which may be an intermediary for the increased adverse neonatal outcomes (Fig. 1). In previous studies on the association between IPI and perinatal outcomes, maternal complications were used as the outcomes, and the influence of maternal complications on neonatal outcomes was ignored [21, 22].

After the implementation of the universal two-child policy in China, more antenatal care and examinations have been strengthened to reduce adverse neonatal outcomes [17]. However, as potentially modifiable risk factors, it is essential to determine the mechanism of IPI and maternal age on neonatal outcomes. Thus, the present study aimed to reveal the comprehensive associations between IPI and maternal age with adverse neonatal outcomes and to provide epidemiological evidence for the formulation of public health policies and prepregnancy consultation for reproductive women.

## Methods

The data were collected from the Maternity and Child Registration System in the present historical cohort study. It was provided by the Health Commission of Luzhou City and used for tracking and managing pregnant women and their fetuses/newborns who visited and gave birth in all hospitals in Luzhou district. After obtaining electronic authorization, the Maternity and Child Registration System could scrape the rough data from the Hospital Information System. The outlier data were filtered automatically, and one of our authors checked it in the original data.

A total of 18,605 births were recorded from October 1st, 2015, to October 31st, 2020. The inclusion criterion was multiparas with two or more pregnancies. Primipara (*n* = 9586) was excluded first. Pregnant women with twins or multiple births (*n* = 394), severe diseases (such as cancer, *n* = 8), received assisted reproductive technology (*n* = 787) during the current pregnancy, and data missing on IPI (*n* = 77) or birth weight (*n* = 84) were excluded. A total of 7669 singleton live births were finally eligible for analysis. This was a historical cohort study, and the protocol was reviewed and approved by the Medical Ethics Committee of the Affiliated Hospital of Southwest Medical University (No. KY2021264). As the data were collected anonymously, informed consent was not required by the patients.

IPI was defined as the interval between delivery and conception of the subsequent pregnancy, which was calculated in months from the date of the last birth to the date of the present birth, minus the gestational age, and a long IPI was defined as ≥ 60 months [10, 23]. PTB was delivered before the 37th completed week of gestation. LBW was defined as birthweight < 2500 g, and macrosomia was birthweight > 4000 g. SGA and large for gestation age (LGA) were birthweight less than the 10th percentile and more than the 90th percentile according to sex-age based on Chinese national growth curves, respectively [24].

The factors that potentially influence the associations between exposure and outcomes were adjusted in the analysis, including maternal age at the first delivery (< 25, 25–29, or ≥ 30), maternal age at the current delivery (< 25, 25–29, 30–34, or ≥ 35), gravidity (2, 3, or more than 3), parity (2 or more), body mass index (BMI) at admission for current delivery (< 25, 25–29.9, or ≥ 30), methods of the last delivery (vaginal delivery or cesarean section), abortion history (yes or no), gestational diabetes mellitus (GDM, yes or no) and pregnancy-induced hypertension (PIH, yes or no). BMI was calculated as weight (kg)/height (m)^2^.

The WHO recommends that the IPI should not be less than 24 months [25] and the American College of Obstetricians and Gynecologists recommends that the optimal IPI is 18 months to 5 years [26]. According to these recommendations, the IPI groups were classified as < 12 months, 12–23 months, 24–59 months, and 60 months or greater, and the IPI of 24–59 months was set as the reference group. The outcomes were described as categorical variables, and the chi-squared test (*χ*^*2*^*)* was used to compare the baseline characteristics and outcomes among the groups with different IPIs.

A series of logistic regression models were used to calculate odds ratios (ORs) and 95% confidence intervals (CIs) of the outcomes. The model fitness was checked using the Hosmer and Lemeshow goodness of fit. In Model 1, the crude OR of each outcome for the IPI was calculated by an unadjusted logistic regression. After checking multicollinearity for the data, maternal age at the first delivery, gravidity, parity, BMI, mode of the last delivery and abortion history were adjusted in a multivariable logistic regression (Model 2). Two other logistic models were used to explore the role of maternal age and maternal complications at the present delivery in the association of IPI and neonatal outcomes.

We applied “relative excess risk due to interaction” (RERI) to evaluate the additive interaction between a long IPI and advanced maternal age. The RERI is defined as RERI = OR_11_ − OR_10_ − OR_01_ + 1. The OR_11_ is in group exposure to both long IPI (1 = exposed, 0 = unexposed) and advanced maternal age (1 = exposed, 0 = unexposed), OR_10_ is in group exposure to long IPI, and OR_01_ is in group exposure to advanced maternal age, compared to the doubly unexposed group, respectively. RERI > 0 and RERI < 0 were regarded as significant positive and negative additive interactions, respectively [27]. Furthermore, to interpret the role of maternal age profoundly, a logistic regression analysis was performed to test the association between maternal age and adverse neonatal outcomes. Statistical analyses were conducted with SAS software 9.4 (SAS Institute, Inc., Cary, NC, USA).

## Results

The differences in maternal characteristics among subgroups with different IPIs are displayed in Table 1. A total of 49.9% of the women delivered after long IPIs (≥ 60 months), and 46.8% of the women with long IPIs were ≥ 35 years old. The pregnant women with different IPIs had significant differences in maternal age at the current delivery (*P* < 0.001) and at the first delivery (*P* < 0.001), gravidity (*P* < 0.001), parity (*P* < 0.001), BMI at admission for delivery (*P* < 0.001), methods of the last delivery (*P* < 0.001), abortion history (*P* < 0.001), GDM (*P* < 0.001) and PIH (*P* < 0.001). Pregnant women with long IPIs were older at delivery, had a more frequent pregnancy history, had a higher BMI, had higher risks of GDM and PIH, and had fewer previous abortion history.

**Table 1:** Differences in baseline characteristics among pregnant women with different interpregnancy intervals

Variables	Total (*N* = 7669)	Interpregnancy interval (mon)				*P*
		< 12 (*n* = 350)	12–23 (*n* = 945)	24–59 (*n* = 2544)	≥ 60 (*n *= 3830)	
Maternal age at current delivery (y)						< 0.001
< 25	602 (7.9)	102 (29.1)	213 (22.5)	258 (10.1)	29 (0.8)	
25–29	2070 (27.0)	152 (43.4)	387 (41.0)	969 (38.1)	562 (14.7)	
30–34	2831 (36.9)	63 (18.0)	286 (30.3)	1036 (40.7)	1446 (37.8)	
≥ 35	2166 (28.2)	33 (9.4)	59 (6.2)	281 (11.1)	1793 (46.8)	
Maternal age at first delivery(y)						< 0.001
< 25	4123 (53.8)	197 (56.3)	479 (50.7)	1222 (48.0)	2225 (58.1)	
25–29	2982 (38.9)	108 (30.9)	356 (37.7)	1081 (42.5)	1437 (37.5)	
≥ 30	564 (7.3)	45 (12.9)	110 (11.6)	241 (9.5)	168 (4.4)	
Gravidity						< 0.001
2	1909 (24.9)	171 (48.9)	360 (38.1)	726 (28.5)	652 (17.0)	
3	2156 (28.1)	96 (27.4)	296 (31.3)	800 (31.5)	964 (25.2)	
> 3	3604 (47.0)	83 (23.7)	289 (30.6)	1018 (40.0)	2214 (57.8)	
Parity						< 0.001
2	6055 (78.9)	255 (72.9)	688 (72.8)	1894 (74.4)	3218 (84.0)	
> 2	1614 (21.1)	95 (27.1)	257 (27.2)	650 (25.6)	612 (16.0)	
Body mass index at admission for delivery						< 0.001
< 25	1948 (25.4)	132 (37.7)	311 (32.9)	653 (25.7)	852 (22.2)	
25–29.9	3890 (50.7)	149 (42.6)	433 (45.8)	1317 (51.8)	1991 (52.0)	
≥ 30	1411 (18.4)	46 (13.1)	139 (14.7)	442 (17.3)	784 (20.5)	
Missing	420 (5.5)	23 (6.6)	62 (6.6)	132 (5.2)	203 (5.3)	
Last cesarean section						< 0.001
Yes	4884 (63.7)	163 (46.5)	545 (57.7)	1702 (66.9)	2474 (64.6)	
No	2749 (35.8)	185 (52.9)	393 (41.6)	829 (32.6)	1342 (35.0)	
Missing	36 (0.5)	2 (0.6)	7 (0.7)	13 (0.5)	14 (0.4)	
Previous abortion history						< 0.001
Yes	5299 (69.1)	133 (38.0)	483 (51.1)	1625 (63.9)	3058 (79.8)	
No	2370 (30.9)	217 (62.0)	462 (48.9)	919 (36.1)	772 (20.2)	
Gestational diabetes mellitus						< 0.001
Yes	924 (12.1)	32 (9.1)	65 (6.9)	235 (9.2)	592 (15.5)	
No	6745 (87.9)	318 (90.9)	880 (93.1)	2309 (90.8)	3238 (84.5)	
Pregnancy-induced hypertension						< 0.001
Yes	534 (7.0)	14 (4.0)	45 (4.8)	126 (5.0)	349 (9.1)	
No	7135 (93.0)	336 (96.0)	900 (95.2)	2418 (95.0)	3481 (90.9)	

Table 2 shows the differences in neonatal outcomes in different subgroups, and there were significant differences in PTB (*P* < 0.001), LBW (*P* < 0.001), and one-minute Apgar score ≤ 7 (*P* < 0.001). Higher rates of PTB, LBW, and one-minute Apgar score ≤ 7 were observed in infants born to mothers with IPI < 12 months or IPI ≥ 60 months.

**Table 2:** Differences in adverse neonatal outcomes among groups with different interpregnancy intervals

Variables	Total	Interpregnancy interval (mon)				*χ*^2^	*P*
		< 12	12–23	24–59	≥ 60		
Preterm birth	1294 (16.9)	82 (23.4)	161 (17.0)	371 (14.6)	680 (17.8)	22.3	< 0.001
Low birth weight	930 (12.1)	64 (18.3)	114 (12.1)	261 (10.3)	491 (12.8)	22.5	< 0.001
Macrosomia	357 (4.7)	13 (3.7)	50 (5.3)	118 (4.6)	176 (4.6)	1.5	0.661
Small for gestational age	419 (5.5)	25 (7.1)	44 (4.7)	128 (5.0)	222 (5.8)	4.8	0.184
Large for gestational age	1373 (17.9)	53 (15.1)	156 (16.5)	470 (18.5)	694 (18.1)	3.7	0.289
1-min Apgar score ≤ 7	342 (4.5)	29 (8.3)	41 (4.3)	92 (3.6)	180 (4.7)	16.8	0.001

In Table 3 Model 2, compared with the reference group (IPI at 24–59 months), the long IPI group (IPI ≥ 60 months) was associated with a higher risk of PTB (adjusted OR, 1.15; 95% CI 1.00–1.34) and LBW (adjusted OR, 1.19; 95% CI 1.00–1.41). Model 3 showed that while entering maternal age at the current delivery, the ORs in long IPIs for PTB, LBW, and one-minute Apgar score ≤ 7 increased. In addition, negative interaction effects were observed between a long IPI and advanced maternal age for PTB (RERI =  − 0.62), LBW (RERI =  − 0.78), and one-minute Apgar score ≤ 7 (RERI =  − 1.35). When maternal age at the current delivery and maternal complications were entered simultaneously in Model 4, the long IPI group was still associated with a higher risk of PTB (adjusted OR, 1.24; 95% CI: 1.04–1.47), LBW (adjusted OR, 1.29; 95% CI 1.05–1.58), and 1-min Apgar score ≤ 7 (adjusted OR, 1.42; 95% CI 1.04–1.94). This indicates that a long IPI is an independent risk factor for PTB, LBW and a 1-min Apgar score ≤ 7.

**Table 3:** Crude and adjusted odds ratios for adverse neonatal outcomes in groups with interpregnancy intervals

Variables	Model 1^a^ crude OR (95% CI)	Model 2^b^ adjusted OR (95% CI)	Model 3^c^ adjusted OR (95% CI)	Model 4^d^ adjusted OR (95% CI)
Preterm birth				
IPI < 12 mon	1.79 (1.37–2.35)	1.65 (1.25–2.19)	1.51 (1.13–2.01)	1.51 (1.13–2.02)
IPI 12–23 mon	1.20 (0.98–1.47)	1.13 (0.92–1.39)	1.06 (0.85–1.31)	1.06 (0.85–1.31)
IPI 24–59 mon	Reference	Reference	Reference	Reference
IPI ≥ 60 mon	1.26 (1.10–1.45)	1.15 (1.00–1.34)	1.27 (1.07–1.50)	1.24 (1.04–1.47)
Low birth weight				
IPI < 12 mon	1.96 (1.45–2.64)	1.67 (1.22–2.28)	1.50 (1.09–2.07)	1.52 (1.10–2.11)
IPI 12–23 mon	1.20 (0.95–1.52)	1.08 (0.85–1.37)	1.00 (0.78–1.27)	1.00 (0.78–1.28)
IPI 24–59 mon	Reference	Reference	Reference	Reference
IPI ≥ 60 mon	1.29 (1.10–1.51)	1.19 (1.00–1.41)	1.32 (1.08–1.61)	1.29 (1.05–1.58)
Small for gestation age				
IPI < 12 mon	1.45 (0.93–2.27)	1.23 (0.78–1.93)	1.13 (0.71–1.79)	1.15 (0.71–1.84)
IPI 12–23 mon	0.92 (0.65–1.31)	0.83 (0.59–1.19)	0.78 (0.54–1.12)	0.77 (0.53–1.11)
IPI 24–59 mon	Reference	Reference	Reference	Reference
IPI ≥ 60 mon	1.16 (0.93–1.45)	1.16 (0.92–1.47)	1.21 (0.92–1.59)	1.18 (0.89–1.56)
1-min Apgar score ≤ 7				
IPI < 12 mon	2.41 (1.56–3.71)	2.13 (1.36–3.32)	1.93 (1.23–3.04)	1.93 (1.22–3.04)
IPI 12–23 mon	1.21 (0.83–1.76)	1.12 (0.76–1.63)	1.03 (0.70–1.52)	1.03 (0.70–1.52)
IPI 24–59 mon	Reference	Reference	Reference	Reference
IPI ≥ 60 mon	1.31 (1.02–1.70)	1.26 (0.97–1.65)	1.46 (1.07–1.98)	1.42 (1.04–1.94)

Model 1: crude OR

Model 2: adjusted for maternal age at first delivery, gravidity, parity, BMI at admission for delivery, last cesarean section, and previous abortion history

Model 3: adjusted for maternal age at first delivery, gravidity, parity, BMI at admission for delivery, last cesarean section, previous abortion history, and maternal age at current delivery

Model 4: adjusted for maternal age at first delivery, gravidity, parity, BMI at admission for delivery, last cesarean section, previous abortion history, gestational diabetes mellitus, pregnancy-induced hypertension, and maternal age at current delivery

*OR* odds ratio, *CI* confidence interval, *IPI* interpregnancy intervals, *BMI* body mass index

The short IPI group (IPI < 12 months) was also associated with an increased risk of PTB (adjusted OR, 1.51; 95% CI 1.13–2.01), LBW (adjusted OR, 1.50; 95% CI 1.09–2.07), and 1 min Apgar score ≤ 7 (adjusted OR, 1.93; 95% CI 1.23–3.04) (Table 3 Model 3).

## Discussion

Our findings showed that short and long IPIs are associated with an increased risk of PTB, LBW and a 1-min Apgar score ≤ 7. In addition, negative interactions exist between a long IPI and advanced maternal age for these neonatal outcomes.

Consistent with previous studies [10, 16, 28–30], the present historical cohort study indicates that a long IPI is an independent risk factor for adverse neonatal outcomes. Many previous studies [11, 15, 31] examined the association between the IPI and neonatal outcomes, focusing on a short IPI. Unfortunately, few studies [10, 16, 28–30] have examined the association between a long IPI and neonatal outcomes. The specific potential mechanism between long IPI and adverse neonatal outcomes is still not clear. Physiological regression hypothesis was proposed in a previous study that pregnancy helps women obtain the capacity of growth support, and the benefit may gradually be lost after delivery if another pregnancy occurs with long IPIs [32].

With the implementation of the universal two-child policy in China in 2015, the number of pregnant women with a long IPI increased with a higher rate of pregnant women with advanced maternal age [20]. In the present study population, almost half of the pregnant women with long IPIs (≥ 60 months) were 35 years or older. Many previous studies [17, 18, 33] have reported that advanced maternal age increases the risk of adverse neonatal outcomes. Interestingly, advanced maternal age was not associated with an increased risk of adverse neonatal outcomes in the present study (Supplementary Table 1), consistent with the report of Qin et al. in 2017 [34]. This phenomenon may be attributed to the self-selection of pregnant women and their family support. In this initial stage of the universal two-child policy implementation, women with an advanced age who would like to bear a second baby might have a better socioeconomic and health status and may even have improved health care during pregnancy.

In contrast to previous studies, the present study aimed to interpret the interactions between a long IPI and advanced maternal age on adverse neonatal outcomes. When maternal age at the current delivery was included in the model, ORs were increased in the long IPI group for PTB, LBW and one-minute Apgar score ≤ 7 (Table 3, Model 3) compared with before adjustment. Furthermore, negative interaction effects were observed between a long IPI and advanced maternal age in the RERI model. Both results support that advanced maternal age does not increase the risk of adverse neonatal outcomes in our study subjects. Thus, we conclude that a long IPI is an independent risk factor for adverse neonatal outcomes, but advanced age does not strengthen this effect in this initial stage of universal two-child policy implementation in China.

In some previous studies, both a long IPI and advanced maternal age were associated with a higher incidence of maternal complications [21, 22, 35, 36], and maternal complications were also proven to correlate with adverse neonatal outcomes [37, 38]. Thus, maternal complications should be considered the intermediate variable in the pathway between a long IPI and neonatal outcomes (Fig. 1). Based on this directed acyclic graph (DAG) and some well-known paradoxes in epidemiology studies [39, 40], an overadjustment bias is generated by adjusting for an intermediate variable [41]. In contrast to maternal age, with controlling for maternal complications, the real associations between a long IPI and neonatal outcomes cannot be consistently estimated. In the present study, the ORs of long IPIs for neonatal outcomes decreased after adjusting for maternal complications (Table 3, Model 4). Thus, the real associations between IPIs and neonatal outcomes can be estimated from Model 3 (Table 3). We should strictly distinguish the interaction and intermediate effect in future studies.

In the present study, a short IPI was also associated with increased risks of PTB, LBW, and a 1-min Apgar score ≤ 7, which is consistent with previous studies [12, 42]. A short interval between successive pregnancies may worsen maternal nutritional status by reducing the time to recover from delivery. Additionally, breastfeeding can enhance maternal malnutrition, leading to insufficient placental function [32]. In the clinical consultation, we should also advise women to avoid pregnancy after a short IPI.

Our study has several limitations. We did not address some residual confounding factors, such as maternal smoking, alcohol, pregnancy intention, maternal illness, and fertility issues. Additionally, we did not include pregnancies induced by assisted reproductive technology. These women have worse fertility and may have an increased risk of adverse neonatal outcomes. Although we included both live births and stillbirths after at least 28 gestational weeks, we did not address pregnancy loss before 28 weeks. Furthermore, we performed a single-center study with the advantage of excluding the bias of different therapeutic approaches in obstetrics that could affect neonatal outcomes; thus, the results may not be generalized to the whole Chinese cohorts.

In conclusion, our data showed that short and long IPIs are associated with an increased risk of adverse neonatal outcomes after implementing the universal two-child policy in China. While planning to give birth to another baby, an appropriate IPI should be recommended to reduce the risks of adverse neonatal outcomes. In addition, better antenatal care might be taken to balance the inferiority of advanced maternal age and to improve neonatal outcomes.

### Supplementary Information

Below is the link to the electronic supplementary material.

Supplementary file1 (DOCX 18 KB)

## Data Availability

From the publication date, upon reasonable request to the corresponding author (researchers who provide a methodologically sound proposal and assuming use of the data to meet the goals of this proposal), individual participant data that underlie the results reported in this article can be made available after de-identification.
