# Supplementary material for: Interactions between long interpregnancy interval and advanced maternal age on neonatal outcomes
Source: World J Pediatr. 2023 Apr 26;19(12):1155–61. doi: 10.1007/s12519-023-00728-4 (PMC10590323; doi:10.1007/s12519-023-00728-4)
Supplement: Supplementary file 1 — Supplementary file1 (DOCX 18 KB) [file 12519_2023_728_MOESM1_ESM.docx]

**Supplementary material:**

Supplementary Table S1. Crude and adjusted odds ratios for adverse neonatal outcomes by maternal age at current delivery

|  | Model 1^a^  crude *OR* (95% *CI*) | Model 2^b^  adjusted *OR* (95% *CI*) | Model 3^c^  adjusted *OR* (95% *CI*) |
| --- | --- | --- | --- |
| Preterm birth | | |  |
| <25y | 1.57 (1.27-1.94) | 1.37 (1.10-1.72) | 1.40 (1.11-1.77) |
| 25-29y | Reference | Reference | Reference |
| 30-34y | 0.75 (0.65-0.88) | 0.90 (0.76-1.06) | 0.85 (0.71-1.01) |
| ≥35y | 0.90 (0.77-1.06) | 1.03 (0.86-1.23) | 0.91 (0.74-1.12) |
| Low birth weight | | |  |
| <25y | 1.71 (1.35-2.16) | 1.43 (1.12-1.83) | 1.50 (1.16-1.94) |
| 25-29y | Reference | Reference | Reference |
| 30-34y | 0.74 (0.62-0.89) | 0.93 (0.77-1.12) | 0.86 (0.70-1.05) |
| ≥35y | 0.87 (0.72-1.05) | 1.07 (0.87-1.32) | 0.92 (0.72-1.17) |
| Small for gestation age | | |  |
| <25y | 1.66 (1.17-2.36) | 1.47 (1.03-2.10) | 1.61 (1.11-2.34) |
| 25-29y | Reference | Reference | Reference |
| 30-34y | 0.96 (0.74-1.25) | 1.13 (0.86-1.48) | 1.03 (0.78-1.38) |
| ≥35y | 1.09 (0.83-1.42) | 1.28 (0.95-1.72) | 1.09 (0.77-1.54) |
| 1-minute Apgar score ≤7 | | | |
| <25y | 1.43 (0.98-2.09) | 1.34 (0.91-1.98) | 1.39 (0.92-2.09) |
| 25-29y | Reference | Reference | Reference |
| 30-34y | 0.79 (0.60-1.05) | 0.86 (0.64-1.16) | 0.79 (0.58-1.08) |
| ≥35y | 0.94 (0.71-1.26) | 0.96 (0.70-1.33) | 0.80 (0.55-1.16) |

a, Model 1: Crude *OR* (95% *CI*).

b, Model 2: Adjusted for maternal age at first delivery, Gravidity, Parity, BMI at admission for delivery, Last cesarean section, and Previous abortion history.

c, Model 3: Adjusted for maternal age at first delivery, Gravidity, Parity, BMI at admission for delivery, Last cesarean section, Previous abortion history, and Interpregnancy interval.
